# The First Physiologically Based Pharmacokinetic (PBPK) Model for an Oral Vaccine Using Alpha-Tocopherol as an Adjuvant

**DOI:** 10.3390/pharmaceutics15092313

**Published:** 2023-09-13

**Authors:** Leonor Saldanha, Nuno Vale

**Affiliations:** 1 OncoPharma Research Group, Center for Health Technology and Services Research (CINTESIS), Rua Doutor Plácido da Costa, 4200-450 Porto, Portugal; leonorpessanha@gmail.com; 2 CINTESIS@RISE, Faculty of Medicine, University of Porto, Alameda Professor Hernâni Monteiro, 4200-319 Porto, Portugal; 3 Department of Community Medicine, Health Information and Decision (MEDCIDS), Faculty of Medicine, University of Porto, Rua Doutor Plácido da Costa, 4200-450 Porto, Portugal

**Keywords:** PBPK, vaccine, oral, alpha-tocopherol, adjuvant, metabolism, immunology

## Abstract

Oral vaccines represent many advantages compared to standard vaccines. They hold a simple method of administration and manufacturing process. In addition to these, the way they can induce immune responses makes these a promising technology for the pharmaceutical industry and represents a new hope to society. Physiologically based pharmacokinetics (PBPK) has been used in support of drug development to predict the pharmacokinetics of the compound, considering the patient’s physiology. Despite PBPK studies now being widely used, there are very few models in the literature that support vaccine development. Therefore, the goal of this article was to determine how PBPK could support vaccine development. The first PBPK model for an oral vaccine using alpha-tocopherol as a vaccine adjuvant was built. LogP is the parameter that influences the delivery of alpha-tocopherol into the tissues more. Having a high LogP means it accumulates in adipose tissue and is slowly metabolized. The ideal formulation to include alpha-tocopherol in an oral vaccine would incorporate nanoparticles in a capsule, and the dosage of the compound would be 150 mg in a volume of 200 mL. This article aims to determine if alpha-tocopherol, as a well-known adjuvant for intramuscular injection vaccines, could be used as an adjuvant to oral vaccines. This model was built considering the conditions and requirements needed for designing an oral vaccine. This implies making sure the antigen and adjuvants reach the main target by overcoming the challenges of the gastrointestinal tract. The main parameters that would need to be included in a formulation using alpha-tocopherol as an adjuvant were determined.

## 1. Introduction

Since the COVID-19 pandemic, vaccines assumed an important position in drug development for many companies [1,2]. The trend to invest in vaccines is not only related to their popularity during and after the pandemic. However, it is a fact that the technological advancements related to innovative vaccine platforms used against coronavirus-19 allowed a technological boost. Additionally, it has been seen as an endorsement of new technologies and the development of stronger cooperation amongst many stakeholders to build relevant guidelines and to gather conditions for an optimal vaccine development path that could lead to success [3,4]. In addition to the above, vaccines are also seen as a new hope for oncology and other rare diseases while they are still the most valuable resource to prevent infectious diseases [5].

According to a recent report from Delloite, 60 executives from life-science companies have been surveyed, and 95% reported the intent to focus on the development of innovative products. A total of 91% mentioned having plans to invest in R&D innovation. It was also reported that the emergence of mRNA vaccines revitalized the R&D portfolios. This is because mRNA is seen as the future of medicine. Vaccines and other advanced therapies, such as cell gene therapy, reflect a new breadth for science but also for life-science companies’ revenue [4]. There is news everywhere about new hires and divisions built by pharmaceutical companies to focus on vaccines. By the end of 2021, AstraZeneca announced the creation of a new division of vaccines and immune therapies. Moderna announced the hire of around 2000 employees by the end of 2023, focusing on mRNA vaccines [6,7]. PwC also reported that it is likely to see market and acquisition activities and biotech deals related to vaccines in the near future [8].

This trend raises the need to explore new ways of contributing to the research and development of vaccines. In silico studies, namely, physiologically based pharmacokinetics (PBPK) and population pharmacokinetics (PoPPK), are used by pharmaceutical companies and have been endorsed by the EMA and FDA. However, according to a recent review, there are very few PBPK models related to vaccines in the literature until this date [9]. One model studied alpha-tocopherol pharmacokinetics as the main component of the AS03 adjuvant [10]. AS03 is composed of α-tocopherol, squalene, and polysorbate 80 in an oil-in-water emulsion [11].

Adjuvants allow a reduction in the dose of the antigen to be used and in the number of needed vaccinations by enhancing the immune response. It also increases the stability of the vaccine. AS03 demonstrated a substantial improvement in immune responses to antigens of the influenza vaccine. These vaccines provided better immune responses than squalene oil-only adjuvanted vaccines in preclinical studies using mice, and the same effect was verified in humans. This allowed the researchers to discover the potential of α-tocopherol as an immunomodulator. α-Tocopherol can also be called vitamin E and is used as an antioxidant in oral drugs and cosmetics [12]. Additionally, due to its antioxidant properties, vitamin E is also being considered for cancer treatment and prevention. Studies have discovered that other isoforms, such as γ-tocopherol, δ-tocopherol, and δ-tocotrienol, have more cancer-preventive properties than α-tocopherol. There is evidence that these isoforms could be effective in cancer prevention or in acting as adjuvants for improving the therapy or the control of cancer [13,14].

The pharmacokinetics and distribution of alpha-tocopherol for IM administration were explored in a previous PBPK study from 2015 [10].

**Figure 1:**
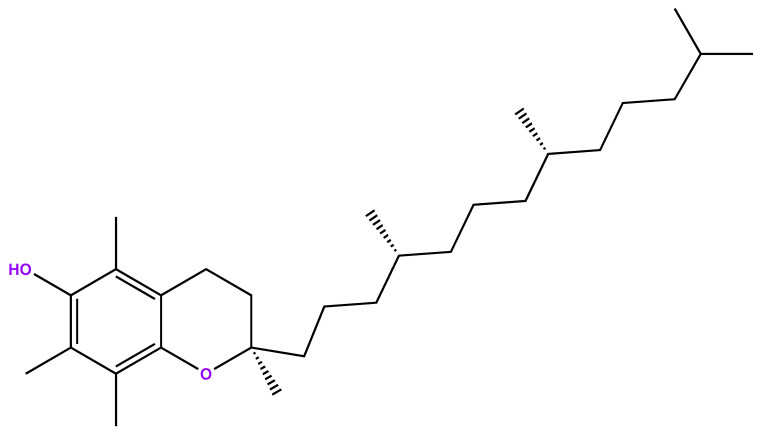
Chemical structure of alpha-tocopherol.

This article intended to build the first PBPK model to support the development of an oral vaccine by analyzing the hypothesis of using alpha-tocopherol (Figure 1) as an adjuvant and the implications required in the formulation, considering the fundamental properties of an oral vaccine. The interest in vaccines administered orally is related to their simple administration and to how local and systemic immune responses are induced [15]. Standard vaccine delivery systems (through injection) reflect issues related to safety and morbidity. They also have a high cost of mass immunization since they require skilled personnel to perform the administration process [16,17]. Additionally, oral vaccines are also easier to manufacture and store in terms of logistics [18].

It is known that the majority of pathogens enter the human body through mucosal sites. Vaccines administered via injection only provide partial or are not able to provide protection at mucosal sites. Oral vaccination could play an important role in overcoming this limitation of standard vaccines by creating immunity at mucosal sides and acting as a barrier against pathogens [16,19]. This is because the humoral and cellular immune responses are enhanced by the oral route, providing longer and wider protection.

Oral vaccines are then a promising technology to facilitate logistics and to overcome the limitations of injected vaccines. However, there are only a few oral vaccines approved [20]. Amongst the approved vaccines, it can be found that the used formulations include live attenuated or whole-cell inactivated vaccines [20,21]. This is due to the main challenges of oral vaccines. To ensure a proper induction of an immune response via the oral route, successful delivery of the active antigen to the intestine is required (it has to be intact), the transport through the mucosal barrier has to be ensured, and the activation of antigen-presenting cells has to be guaranteed. The GI tract environment is the biggest challenge that needs to be overcome in order to achieve the proper induction of the immune response. It may interfere with the integrity of the antigen due to the degradation of the antigens. The pH varies across the GI, ranging from an acidic environment (stomach) to a slightly acidic or neutral environment (pH 6–7.4) in the intestine, and includes secretions of enzymes and bile salts for digestion [22].

The epithelial cell surface is also compact and includes a thick mucus layer that limits the absorption of antigens and their time in the intestine [22]. Considering all these barriers, oral vaccines need to include a larger dose of antigens to obtain the desired mucosal immune responses when compared with standard vaccines. However, this can lead to tolerance. Therefore, it is important to have adequate doses to generate immunity instead of tolerance. Immune tolerance is induced by T cells and can prevent the generation of active immune responses and cause the inflammation of the intestinal tract to induce intestinal inflammatory diseases. All of these aspects need to be considered in the vaccine design [15].

The intestinal epithelia consist of different cells such as enterocytes, microfold cells (M cells), and goblet cells. These are fundamental to vaccine penetration and absorption. Enterocytes represent 85% of the cells in the small intestine and are responsible for the absorption and transport of antigens across the epithelial layer to the mucosa-associated lymphoid tissue (MALTs). There are also tight junctions (TJs) that tightly connect the enterocytes to form a solid barrier to avoid the invasion of the small intestine. M cells can be found in the epithelial layer of intestinal Peyer’s patches (PPs) and perform antigen transcytosis and initiation of immune responses. Goblet cells consist of 15% of the total cells in the small intestine; by secreting mucins, they play an important role in inhibiting the penetration of antigens through the mucosal layer [15]. All of these cells can be considered targets for oral delivery systems for oral vaccines due to their key role involved in penetration, absorption, transport, and immunity. M Cells are a common target for delivery vehicles incorporated in oral vaccines [18].

Oral vaccines may be able to reach these targets depending on the property’s formulation and particle size. For instance, M cells can capture materials such as viruses and bacteria, and this uptake can be improved by turning the particles lipidic in nature or by placing ligands on their surface that are recognized by M cells [15].

Particle size may also have an impact on targeting these cells and in reaching the immune sites capable of inducing immune responses [18]. Nanoparticles (NP) may support the oral delivery of vaccines by enhancing permeation in the intestine through the opening of tight junctions, which will avoid the requirement of cellular uptake and transport across the epithelial barrier. In other cases, there is still a need for endocytosis/exocytosis dynamics to cross the GI tract, and size remains an important parameter. For certain cases, a large area of NP can increase interactions with the GI tract after oral delivery. The range between NP 20 and 100 nm focuses on enterocytes take up and the range of 100–500 nm for M cells to transport NP in the GI. The shape of NP is also important [16,23].

Alpha-tocopherol is considered a Class II drug in the biopharmaceutical classification system (BCS), which means that it has low solubility and high permeability. BCS is an important tool used to classify drugs based on certain properties, such as water solubility, dissolution, and intestinal permeability, which will impact the absorption of active substances from oral forms [24,25]. The molecule has a hydrophobic nature, is very lipophilic, and is practically insoluble in water (<0.1 mg/mL) (Ph. Eur., 2001 [26,27]. Therefore, the LogP for alpha-tocopherol is very high. In the literature, it ranges from 9 to 12 [10,28].

Due to these characteristics, finding the right oral formulation for this compound has been a challenge. The alcohol form of a-tocopherol is the one used in influenza vaccine adjuvants, and pharmacokinetics has not been studied experimentally. This is because it is not a regulatory requirement to submit clinical pharmacokinetic studies for vaccines [29]. Despite the challenges related to the compound characteristics, it is reported in the literature that alpha-tocopherol has a high bioavailability of around 80% [30,31].

This study is intended to study the PK properties of alpha-tocopherol when administered orally and how the formulation can be optimized to successfully integrate the drug delivery of an oral vaccine.

## 2. Materials and Methods

### 2.1. Development of the PBPK Model

Since the focus of this model was to explore the oral drug delivery route, GastroPlus^®^ v9.8.3 (Simulation Plus Inc, Lancaster, CA, USA) was the selected computer software to prepare the PBPK model. The GastroPlus^®^ PBPK modeling and simulation platform has been used widely by companies across various industries and departments since 1998 at a commercial level. The software allows the simulation and prediction of pharmacokinetics (PK) and pharmacodynamics (PD) parameters upon the administration of a certain compound or formulation in general. It mainly consists of three fundamental tabs for which researchers can input parameters: compound tab (related to compound characteristics such as physicochemical data); formulation tab (data related to solubility, particle size, and permeation); physiological tab (gut physiology, select fasted vs. fed state); and pharmacokinetics tab (edit PBPK model, input PK parameters) [32,33]. Studies have shown the utility of PBPK methodology for the prediction of human pharmacokinetics and that GastroPlus^®^ endorses a solid experience with the oral route. Additionally, these studies can be performed in different stages of drug development to support the clinical trial design and to understand the dynamics between the compound and formulation [34].

GastroPlus^®^ is divided into several modules for easy management of the platform and for companies to subscribe to only the modules they need on a certain project. For this study, we used the ADMET Predictor, PBPK, and metabolism and transporter modules [33]. Table 1 below explains the functions of the modules that have been used [33].

As already described, alpha-tocopherol has been used as an adjuvant for intra-muscular injections. The adjuvant system in which it is integrated, AS03^®^, is composed of an oil phase emulsified by polysorbate 80 (surfactant). The oil phase has squalene and alpha-tocopherol [11].

The PBPK model previously developed by Million A. Tegenge and Robert J. Mitkus in 2015, focused on the IM route of alpha-tocopherol, will be used to validate our model for the oral route since AUC values will be compared [10]. The AUC represents the integral of drug concentration with respect to time, and it provides valuable information about drug absorption, distribution, metabolism, and elimination. Comparing AUC values is crucial in pharmacology as it helps assess the overall exposure and effectiveness of different drugs or formulations, as comparing drug formulations or dosage regimens, comparing different drugs, assessing drug–drug interactions, predicting therapeutic effects, bioequivalence studies, and pharmacokinetic studies [35,36,37,38,39,40,41,42,43,44].

#### 2.1.1. Compound Characteristics Tab

Understanding the compound characteristics is the most important step for the construction of a PBPK model. Even if there are no experimental data to include in Gastroplus^®^, we could only determine if the software was performing acceptable predictions if we had in-depth knowledge about the compound properties. At least, a theoretical validation could be possible to perform.

Alpha-tocopherol is considered a Class II compound in terms of BCS classification, which means that it has low solubility and high permeability [24]. This is in line with the experimental data found in the literature, namely having a high LogP and low particle size. Experimental data that have been inputted into the software are described in Table 2 below.

Due to a lack of information in the literature, the software prediction values for the clearance, distribution volume, and half-life were used and calculated. Please see Section 3.1.2).

It is important to note that the value of Fup assumed by the software (an adjusted value) is very different from the experimental Fup value. After the adjustment by the software, the values have a very significant decrease. The software recommends the use of this adjusted plasma FUP. The Fup was calculated using the default calculation v9.5 provided by the PBPK module of the software. It assumes an instant equilibrium between unbound neutral concentrations in plasma, extracellular, and intracellular water and, therefore, updates the ratios of unbound concentrations regardless of the partition coefficient (Kp).

**Table 2:** Experimental data of alpha-tocopherol.

Parameter	Value	Source	Notes
BCS Classification	Class II: low solubility, high permeability	[24]	
Molecular weight (g/mol)	430.71	[45]	
LogP	9.8	ChemDraw predicted (v21.0)	
pKa	10.8 (strongest acidic) −4.9 (strongest basic)	[46,47]	
Solubility (mg/mL) vs. pH	insoluble in water Water solubility 1.13 × 10^−4^ mg/mL (ADMET predictor value on water solubility)	[46]	ADMET predictor value was kept
Drug particle density (g/mL)	0.950 at 25 °C/4 °C	[46]	
Particle size (radius—μm) or particle size distribution (d10, d50, d90)	Diameter 0.044; radius: 0.022	[48]	
Peff (cm/s × 10^−4^) or other permeability data	Peff 0.8484 (Caco-2 permeable)	[49]	
Enzyme: Name: CYP4F2 Location (liver mainly, GIT and kidney) Vmax CYP (μM/h/Kg) Sheep—656.4 Human (adult model)—906.0 Human (infant model)—204.0 Km CYP (μM): 42—Rat/in Vitro		[10]	
% Unbound to plasma protein (Fup)	Experiemental data: 12 (0.12) ADMET predictor value: 1.722 × 10^−6^	[10]	ADMET predictor parameter was used since the program calculations did not allow the use of the experimental data. Value had to be adjusted
Blood to plasma concentration ratio	0.3–0.6 (rats)—0.5 was included as estimative	[50]	Used 0.5

##### Selection of the Dosage Form

As explained above, the PBPK model prepared in 2015 for alpha-tocopherol via IM will be used to validate our model.

Together with a study from 1993 that studied the PK of alpha-tocopherol in humans after a single oral administration using capsules as an oral form, potential dosage calculations for our study were performed. Table 3 refers to the literature references stated above that contributed to the calculations, and Table 4 refers to the calculations.

It is important to note that similar patient characteristics to the PBPK IM model were used: white American human adult (male mean body weight, BW = 73 kg, healthy). This is important because age, gender, ethnicity, and disease state of patients may determine the dose adjustment of alpha-tocopherol for it to be effective. These will be essential for the effective dose and frequency of administration determination of the vaccine adjuvant [51,52].

Due to the high viscosity of alpha-tocopherol, it is also a challenge to determine the best oral pharmaceutical form [29]. It is known that these types of compounds may benefit from encapsulation. This process enhances the delivery through improved absorption of poorly water-soluble drugs. The capsules may be hard or soft, depending on their components. The selection of the most appropriate capsule will depend on the formulation and on the compound’s characteristics (viscosity, temperature at which formulation is filled). New manufacturing processes and technology have allowed a better and easier encapsulation process [53].

**Table 3:** The literature references used for dose calculation.

Dosage Form	Dose	AUC	Source
IM Injection	11.86 mg	15.38 μg·h/mL (adult) 96.98 μg·h/mL (infant)	A first-generation physiologically based pharmacokinetic (PBPK) model of alpha-tocopherol in human influenza vaccine adjuvant [10].
Capsules	800 mg	649 ± 69.5 μg·h/mL	Pharmacokinetics and bioavailability of the RRR and all racemic stereoisomers of alpha-tocopherol in humans after single oral administration [54].

**Table 4:** Dosages calculations.

Alpha-Tocopherol	MM (g/mol)	n (mol)	V (mL)	V (L)	Concentration (M) (or mol/L)
	430.7				
Dose 11.86 mg		0.0275	1.0	0.0010	27.54
Dose 100 mg in 250 mL of water		0.2322	250.0	0.2500	0.9287
Dose 200 mg in 100 mL of water		0.4644	100.0	0.1000	4.644
Dose 500 mg in 50 mL of water		1.1609	50.0	0.0500	23.218
Dose 593 mg in 50 mL of water		1.3768	50.0	0.0500	27.54
Dose 800 mg in 68 mL of water		1.8574	68.0	0.0680	27.32

#### 2.1.2. PBPK Model Steps

**Figure 2:**
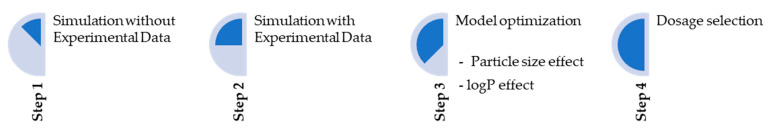
Steps related to the building of the PBPK model.

The flowchart shown in Figure 2 below demonstrates the steps involved in the building of the model. The PBPK model was built for a 30-year-old American man weighing 73 kg in order to keep this model comparable to the PBPK model from Teenage et al. (2015) [10].

- Step 1: PBPK model preparation without experimental data included (ADMET predictor values only).
- Step 2: PBPK model preparation with experimental data included (ADMET predictor values and the literature data). For this step, we included the experimental data detailed in Table 2 above.
- Step 3: Model optimization.
- Step 4: Dosage selection.

As described below, according to the BCS classification, solubility is a limitation for this compound. This has been verified in simulations and discussed in Section 3 below. As a result, model optimization had to focus on the increase of solubility. For this, we tested parameters that can directly interfere with solubility, namely:

(a) Particle Size (Nanoparticles)

Particle size can interfere with solubility and can also overcome some barriers to oral vaccine delivery systems. Although the value found in the literature of ~44 nm of particle size for alpha-tocopherol reflects that it already has a small value, we tried to optimize the value by using the minimal recommended particle size value of nanoparticles for oral vaccines. Higher values of nanoparticles up to 200 nm are within the range for the purpose of being integrated with oral vaccines. However, it did not make sense to test since this compound has very low solubility, and the higher sizes would compromise solubility.

Nanoencapsulating can improve the absorption of alpha-tocopherol by protecting it against the external environment. It has been reported that consuming alpha-tocopherol with lipids can also improve its bioavailability. This was not tested. It seems as well that there is some degree of interindividual variability of alpha-tocopherol bioavailability related to genetic polymorphisms. This is still being researched [55,56].

For oral vaccines, nanoparticles associated with adjuvants have shown enhanced antigen absorption and improved immune response via oral routes by overcoming key barriers of mucosal delivery. For this, the mechanisms related to the transport of the nanoformulations and their physicochemical properties are key factors in reaching an efficient oral vaccine delivery [15].

(b) Reduced LogP (Improve LogP of the Formulation)

LogP has been widely used in drug development to assess the utility of a solute as a drug. In order to assess if a new molecule is a good candidate for the oral route, logP should be between 0 and 5. LogP is the log of the partition coefficient of a solute between octanol and water at near-infinite dilution and is, therefore, also defined as the ratio of the concentration of the unionized compound at equilibrium between organic and aqueous phases [57,58]. The higher the lipophilicity, the less soluble is the compound in water. However, lipophilic compounds usually appear too soluble in oils and lipids and can be good candidates for lipid-based formulations.

Lipophilicity can interfere with solubility, permeability, potency, selectivity, absorption, distribution, metabolism, and excretion (ADME). High lipophilicity is linked to a higher metabolic turnover, low solubility, and diminished oral absorption. An interesting analysis of patented compounds between 2000 and 2010 from 18 well-known pharmaceutical companies, such as AstraZeneca, Lilly, and Novartis, reported that the mean LogP of these patented compounds ranges from 3.5 to 4.5 [58]. Since the LogP of alpha-tocopherol is very high (see Table 2), it compromises the ADME properties of the compound. This step aimed to verify if decreasing LogP to those ideal values for oral drug delivery, as reported in the literature, could improve the PK profile of the compound, namely ADME properties, in order to act as an adjuvant for an oral vaccine. In this study, it was initially planned to run simulations using LogP values between 3.5 and 4.5 and then adjust to other values if needed. However, it was noticed that values of LogP < 6 decreased bioavailability to minimal levels. The LogP of 5 reflected 30% of bioavailability. When increasing to LogP of 5.5, bioavailability increased to 60%, and when increasing to LogP of 6, bioavailability increased to 82%, being the value of bioavailability of alpha-tocopherol reported in the literature (Table 2). According to these results, it was assumed that the LogP of 6 was the ideal value of the compound to increase its solubility and improve its ADME properties.

Now that we had optimized the particle size and the LogP of the compound, the last step was to find the right dosage by performing simulations with the dosages outlined in Table 4. Simulation Time: Simulations were obtained assuming 240 h. This is to make the model comparable with the model from Teneage et al., which also considered 240 h for simulations [10].

#### 2.1.3. PBPK Model Validation

The PBPK model from Tenance et al. 2015 was used to validate and compare our model. AUC can be used to compare different formulations or dosage regimens of the same drug. When we have two different formulations of a drug or excipients, comparing their AUC values can indicate which formulation results in a higher or more sustained drug exposure. Similarly, comparing AUC values for different dosing schedules (e.g., once daily vs. twice daily) can help determine which regimen provides better therapeutic coverage.

## 3. Results

The results for each PBPK model configuration step and respective interpretation can be found below. From the first to the third step, the same dosage and formulation have been used: tablet, IR, 100 mg, volume 250 mL. These are the GastroPlus^®^ standard values for this dosage form.

### 3.1. Results for the PBPK Model by Steps

#### 3.1.1. Results with and without Experimental Data

**Figure 3:**
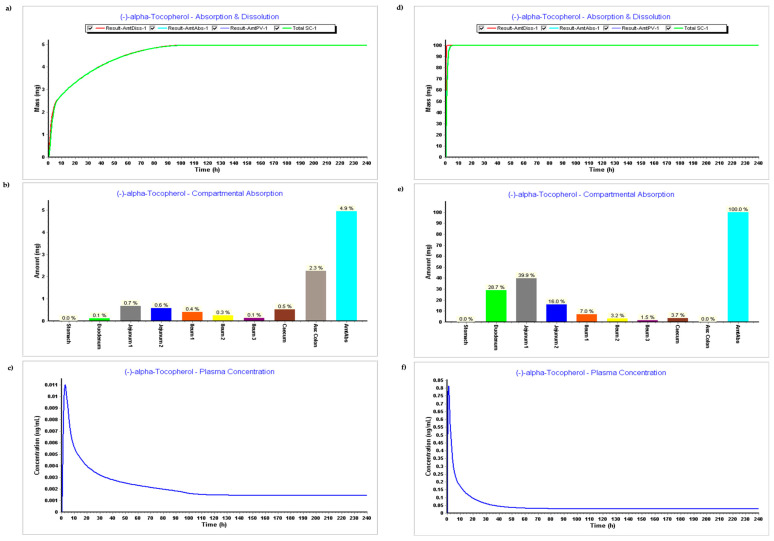
Simulation results during 240 h. (**a**) Absorption and dissolution profile without experimental data (ADMET predictor only); (**b**) Compartmental absorption without experimental data (ADMET predictor only); (**c**) Plasma concentration without experimental data (ADMET predictor only); (**d**) Absorption and dissolution profile with experimental data (ADMET predictor only); (**e**) Compartmental absorption with experimental data (ADMET predictor only); (**f**) Plasma concentration with experimental data (ADMET predictor only).

The results of pk parameters from simulations without experimental data included (ADMET Predictor values only) and with experimental data can be seen in Figure 3 below. These correspond to Steps 1 and 2, respectively.

The results related to the first step (PBPK model without experimental data) can be found in Figure 3a–c, and results related to the second step (PBPK model with experimental data) can be seen in Figure 3d–f.

Dosage Form: IR tablet, 100 mg, volume 250 mL

The absorption and dissolution profile with data predicted by ADMET predictor only based on the secondary structure of the compound, Figure 3a, demonstrates that only 5 mg of the 100 mg were dissolved and absorbed. This might be related to the higher value of the LogP estimated by the ADMET Predictor, version 11^®^. The software module estimated a LogP 12, and LogP input from experimental data was 9. Figure 3b corroborates the fact that there was basically no absorption on the model when the experimental module was not inserted, while Figure 3e demonstrates an absorption of 100%, which seems also to be not accurate since it does not reflect metabolism and distribution.

When comparing Figure 3c,f with plasma concentration during time, we can verify that all parameters such as Cmax, Tmax, and AUC are higher when experimental data are added. Parameters resulting in plasma concentration after simulations with and without experimental data can be found in Table 2 below.

#### 3.1.2. Model Optimization: Particle Size Effect (Step 3)

##### PARTICLE SIZE EFFECT—Testing Minimum Particle Size Allowed and Recommended—Particle Size of 20 nm—Radius: 10 nm

Simulation results after reducing the particle size showed no impact in terms of dissolution profile. As can be seen in Table 2, all PK parameters remained approximately the same with the exception of AUC_0–t,_ which improved from 11.782 to 14.487 μg·h/mL. Considering the above, this particle size was considered an optimization. However, the metabolism and distribution are still not appearing in the model. As a further step of optimization, it was necessary to verify if LogP was influencing these results. It is important to highlight that one of the limitations of this model is that there are no data in the literature about alpha-tocopherol clearance in human adults and that during steps 1 and 2, the ADMET predictor module predicted a CL of 0.242 L/h, a volume of distribution of 33,332.433 L, and a T_1/2_ of 9548 h. This means that the compound was being held forever in the organism and was not being metabolized and excreted.

After reducing the particle size, during Step 3, CL increased to 0.867 L/h, the volume of distribution decreased to 2298 L, and T_1/2_ decreased to 1837 h. There is a slight improvement in metabolism data, but these are still very high values that do not represent reality. Table 5 below compares the results of Steps 1 and 2 and the particle size effect, which is part of the optimization step (Step 3).

##### LogP Effect

Since compartmental absorption was kept the same as in the previous step, the respective graphs were not considered.

- LogP 3.5 vs. LogP 4.0;
- LogP 4.5 vs. LogP 5.0;
- LogP 5.5 vs. LogP 6.0.

##### PARTICLE SIZE 20 nm + LogP 6

Table 6 compares the results of PK parameters with different LogP values applied in the model, separate and together with the particle size effect.

It can be seen that the bioavailability (F%) rises with the rise of LogP. LogP 6 demonstrates a bioavailability of 82%, which is the value reported in the literature. Cmax and Tmax are not impacted, but AUC also increases with the increase of LogP. When particle size is reduced to 20 nm on LogP 6, it can be verified that it does not impact the PK parameters. A summary of clearance, distribution volume (Vss), and half-life time (T_1/2_) of alpha-tocopherol through Steps 1–3 is described in Table 7.

##### Step 4—Dosage and Pharmaceutical Form Finding—Test Different Dosages to Get AUC ~15—PARTICLE SIZE 20 nm + LogP 6

IR Capsule 100 mg volume 250 mL vs. IR Capsule 150 mg volume 200 mL (0.1 h stomach transit time)

##### IR Capsule 593 mg Volume 50 mL vs. IR Capsule 800 mg Volume 68 mL

Table 8 shows the results of the PK parameters when simulating the different dosages and oral forms.

The aim of this step was to verify the best dosage to be used for this formulation. According to the table, the 150 mg capsule in 200 mL would be an ideal candidate since it achieves an AUC_0–inf_ (μg·h/mL) of almost 15 and an AUC_0–t_ (μg·h/mL) of 12.5. This is the closest AUC found in the PBPK model developed for the IM injection found in the literature and validates this dosage.

## 4. Discussion and Conclusions

The results from Step 1 and Step 2 are very important. Step 1 demonstrates the software’s capability to predict certain physiological and physico-chemical properties (such as LogP, pKa, solubility, and permeability). When no experimental data were added (Step 1), results demonstrated that there was basically no absorption. When experimental data were included, the absorption rose to 100%. This should be related to LogP predicted by the software, and the LogP found in the literature. The lower LogP of 9.8 from the literature allows the compound to be absorbed. This is because compounds with extremely high LogP values are very lipophilic. Some lipophilicity may enhance some drug properties, but when LogP and lipophilicity are extremely high, this will lead to issues in drug development due to PK. Water solubility will be compromised, and this will limit dissolution after the drug is administered orally; therefore, absorption will also be compromised [59]. This means that a high LogP will reflect a poor solubility of this compound in the aqueous fluids on the GI tract. Furthermore, these compounds will certainly bind to undesired targets that are hydrophobic and will have issues crossing polar and hydrophilic surfaces of epithelial cells in GI. This may even increase toxicity [60].

As such, LogP is here identified as a very sensitive parameter, and this is why, when designing a drug product for these compounds with high LogP, it is certain that a special formulation or a special excipient will need to be added in order to improve the drug delivery. Furthermore, the distribution and accumulation of these kinds of compounds in fat tissues may potentially lead to toxicity in these tissues. This accumulation and the potential limited access to metabolizing enzymes due to extremely high lipophilicity are related and may lead to poor metabolism and clearance-promoting toxicity. As already reported, most oral drugs have a LogP between 3.5 and 4.5 to ensure optimal ADME properties.

Within the results, it is also possible to verify that there is an absorption of 100%. This demonstrates that something is wrong with the metabolism or distribution. It is important to highlight that one of the limitations of this model is that there are no data in the literature about alpha-tocopherol clearance in human adults and that during Step 1 and 2, the ADMET predictor module predicted a clearance of 0.242 L/h, a volume of distribution of 33,332.433 L, and a T_1/2_ of 9548 h. This means that the compound was being held forever in the organism and was not being metabolized or excreted. This can be seen in Table 7. This is consistent with the information that a high LogP may impact the metabolism as well.

Therefore, after testing some parameters, it was confirmed that LogP was the most sensitive parameter and that it was responsible for this value. When it changed, clearance, volume of distribution and t1/2 also changed. In Table 2, it is possible to verify that parameters such as Cmax, Tmax, and AUC are higher when experimental data are added. Again, being the most sensitive parameter, LogP impacts this value, and the pKa and the particle size contributed to these changes.

Since particle size is a very important parameter in oral vaccine delivery systems and since this formulation will need support to enhance the delivery of alpha-tocopherol due to its high LogP, we tested the lowest value allowed by the software (20 nm), which fits the range of the size provided in the literature. The range between NP 20 and 100 nm focuses on enterocytes taking up to transport NP in the GI. Testing higher values did not make sense since the limitations of this compound are its lipophilicity and solubility issues, and testing larger sizes would not benefit the formulation [16,23].

Since our model also reflected a small particle size of 44 nm according to the information in the literature, when we decreased it to 20 nm, it did not influence the dissolution profile. However, CL increased to 0.867 L/h, the volume of distribution decreased to 2298 L, and T_1/2_ decreased to 1837 h. There is a slight improvement in metabolism data, but these are still very high values that do not represent reality. AUC also improved, and due to this progress, the 20 nm particle size was considered an optimal value for our model.

**Figure 4:**
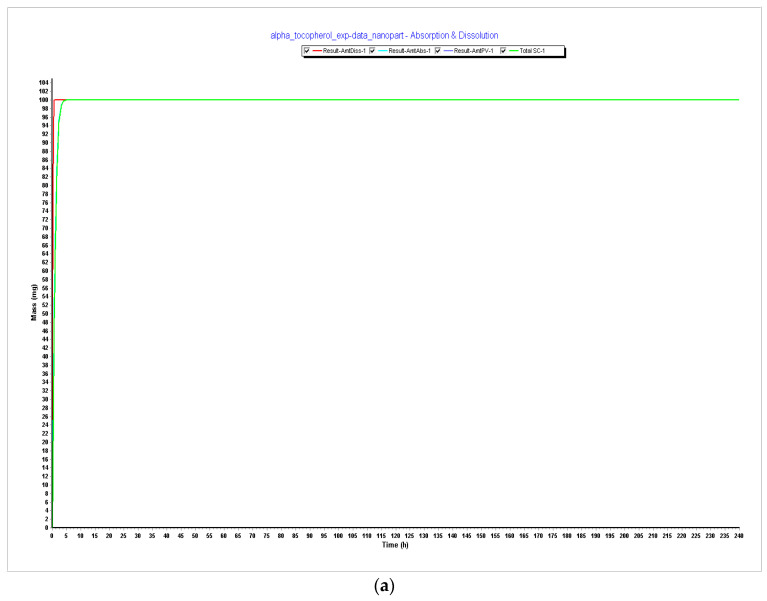

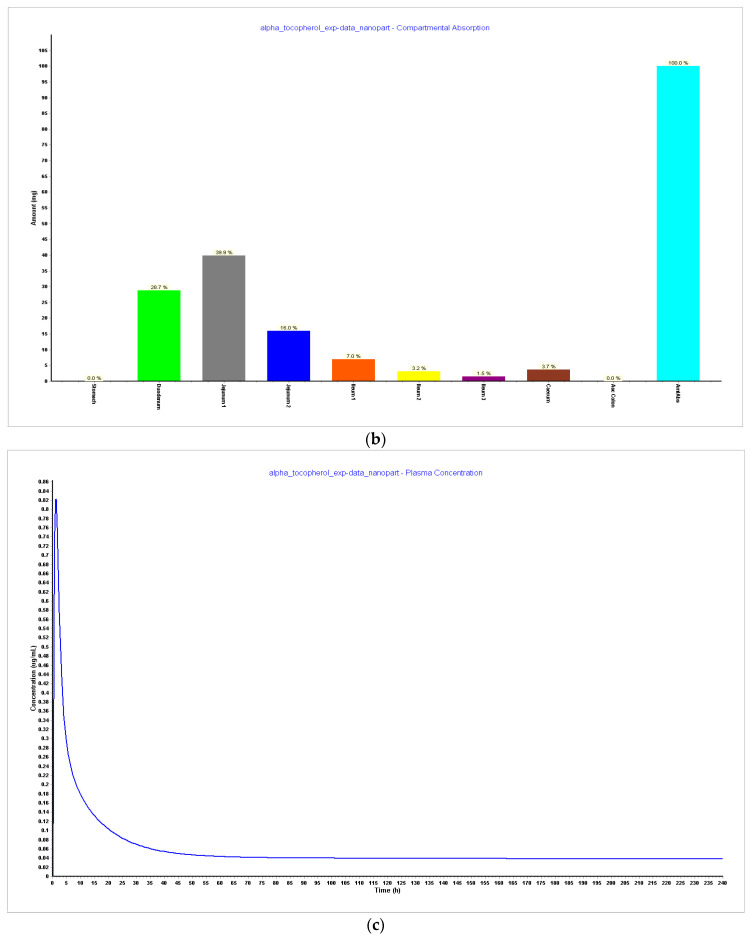
Simulations results during 240 h. (**a**) Absorption and dissolution profile with experimental data and reduced particle size of 20 nm (radius 10 nm); (**b**) compartmental absorption with experimental data and reduced particle size of 20 nm (radius 10 nm); (**c**) plasma concentration with experimental data and reduced particle size of 20 nm (radius 10 nm).

**Figure 5:**
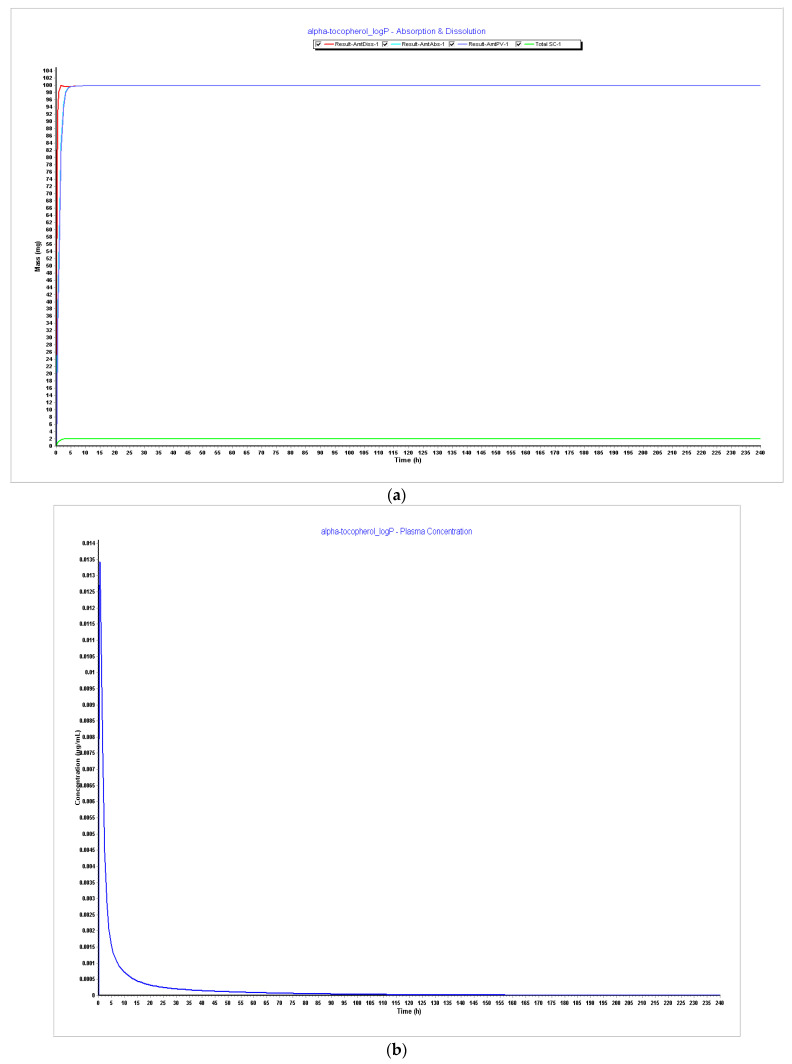

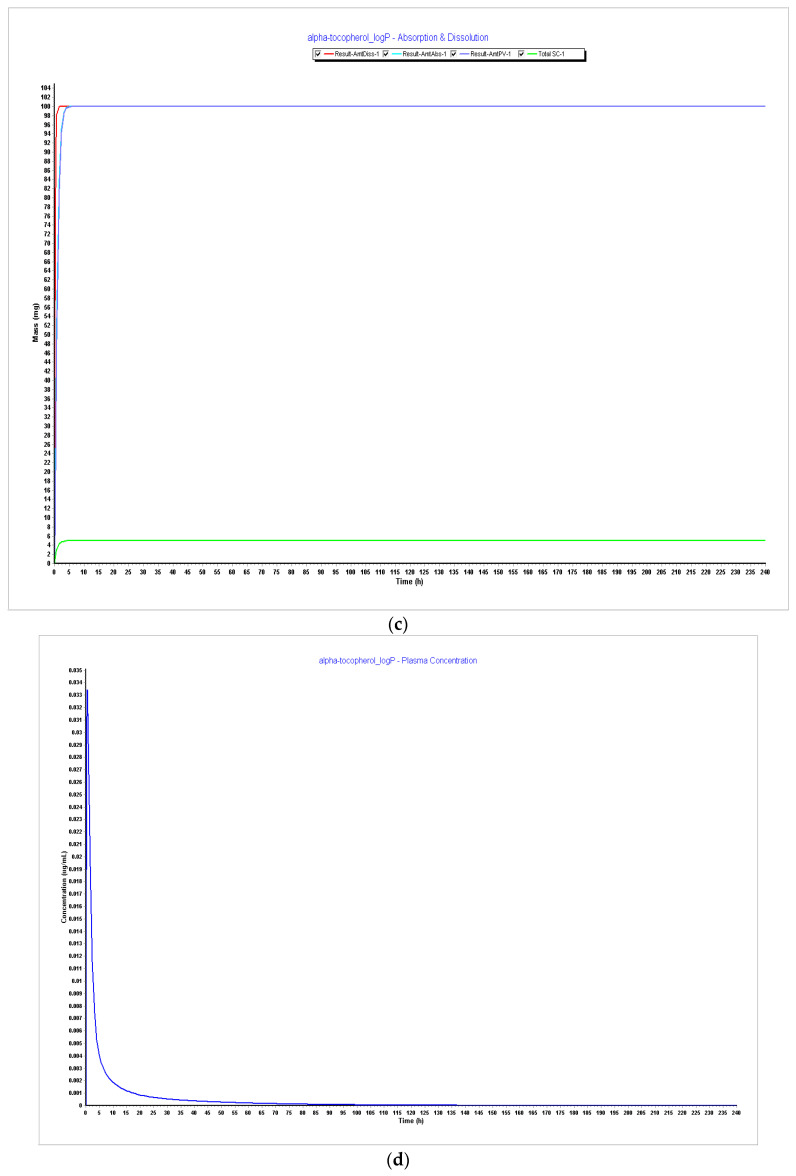
Simulations results during 240 h. (**a**) Absorption and dissolution profile with experimental data and LogP 3.5; (**b**) plasma concentration with experimental data and LogP 3.5; (**c**) absorption and dissolution profile with experimental data and LogP 4.0; (**d**) plasma concentration with experimental data and LogP 4.0.

**Figure 6:**
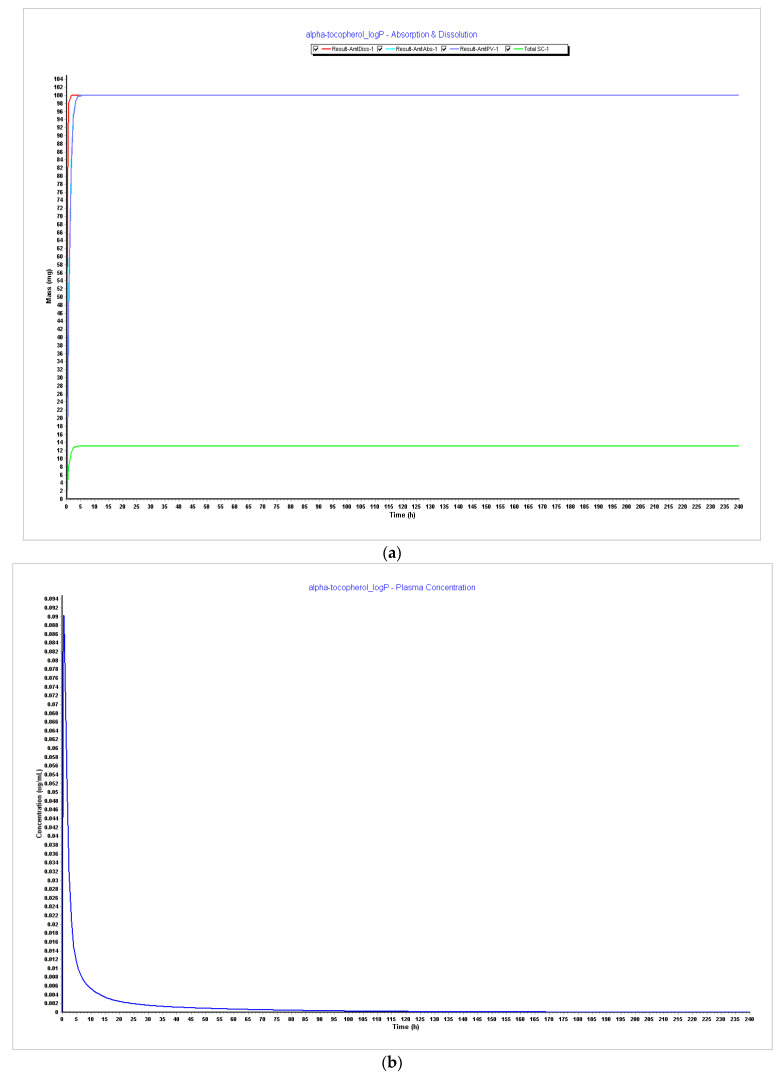

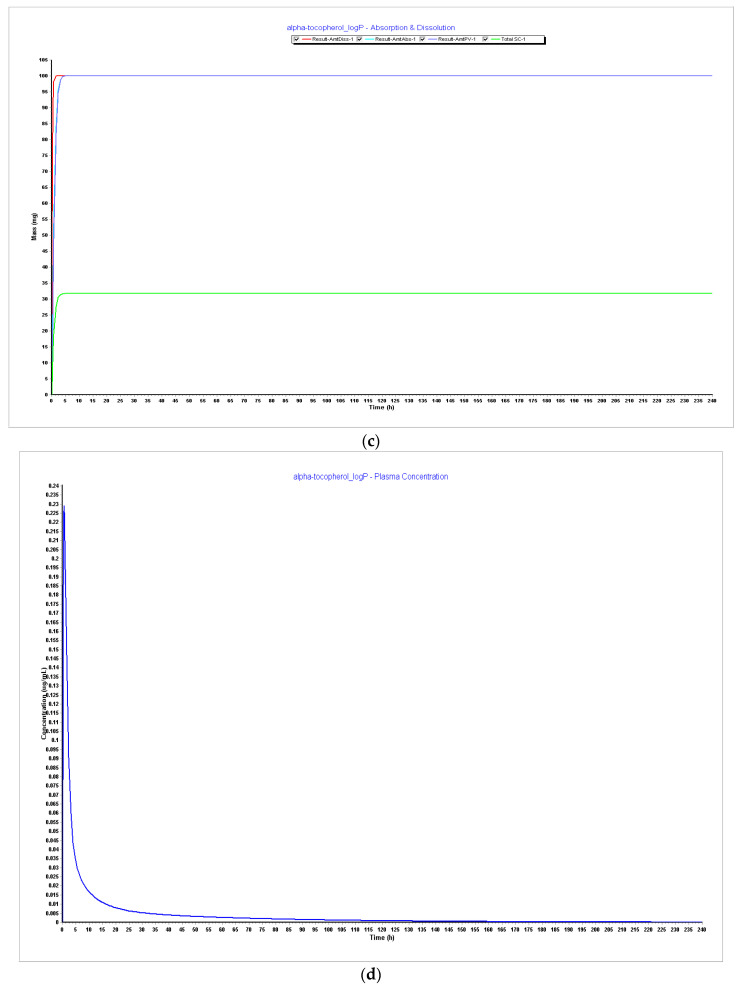
Simulations results during 240 h. (**a**) Absorption and dissolution profile with experimental data and LogP 4.5; (**b**) plasma concentration with experimental data and LogP 4.5; (**c**) absorption and dissolution profile with experimental data and LogP 5.0; (**d**) plasma concentration with experimental data and LogP 5.0.

**Figure 7:**
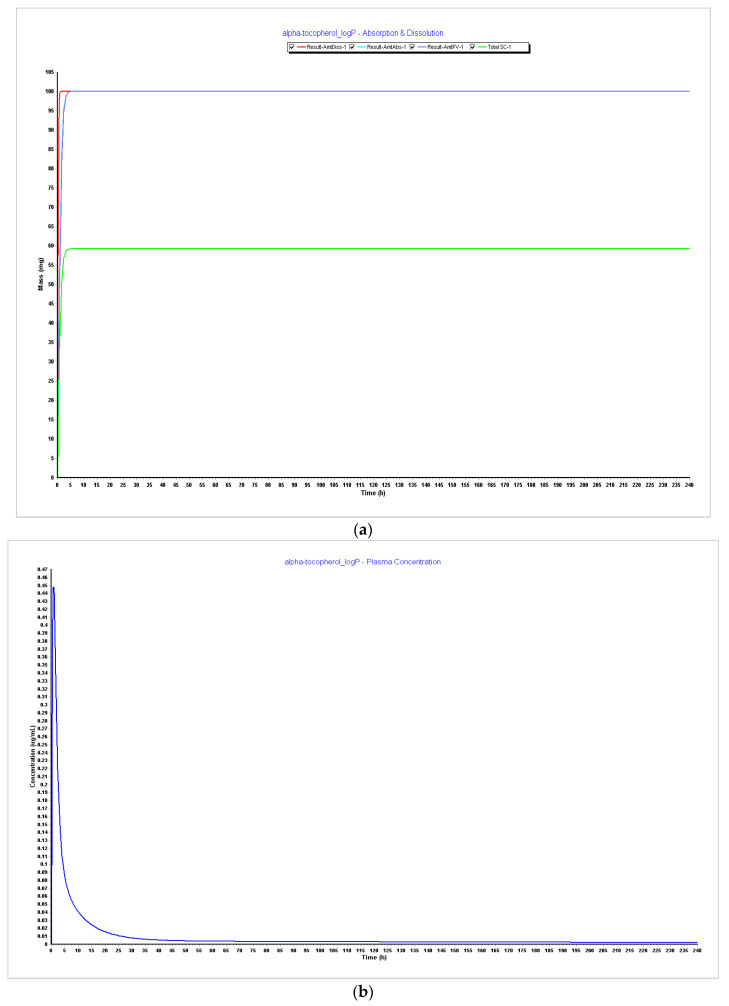

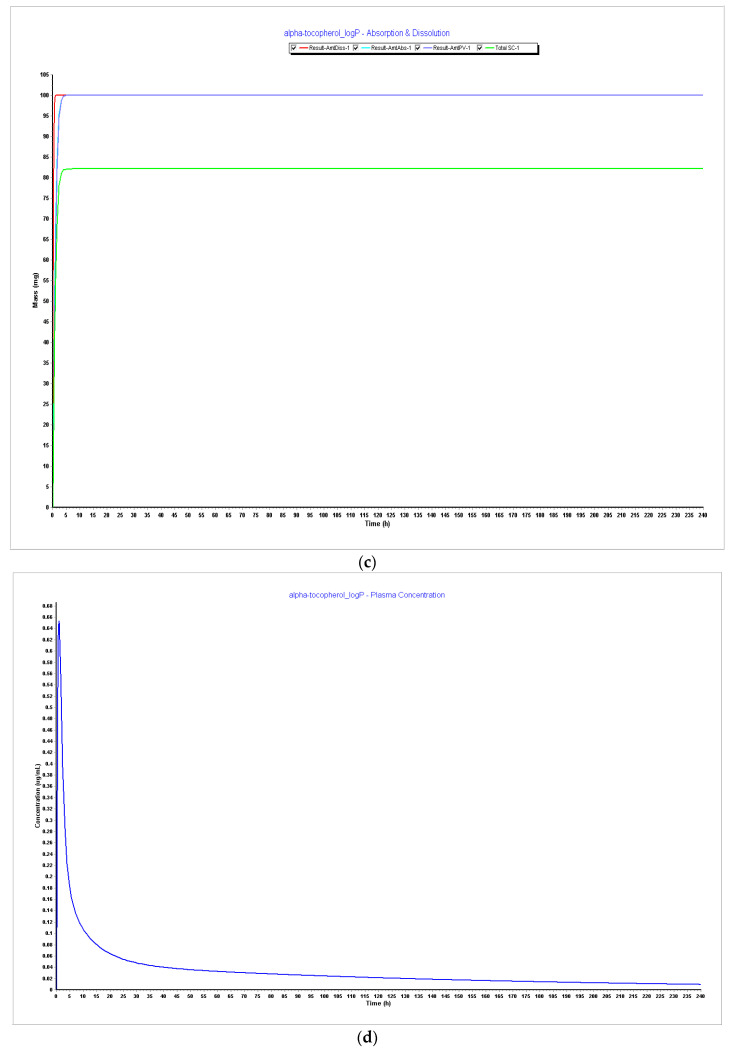
Simulations results during 240 h. (**a**) Absorption and dissolution profile with experimental data and LogP 5.5; (**b**) plasma concentration with experimental data and LogP 5.5; (**c**) absorption and dissolution profile with experimental data and LogP 6.0; (**d**) plasma concentration with experimental data and LogP 6.0.

As a further step of optimization, it was necessary to verify LogP’s impact on the model, considering the information found in the literature about the optimal range of the value being 3.5–4.5 for proper oral delivery and also the hypothesis of this influencing the metabolism and distribution of the compound. As we can see in Figure 4, Figure 5, Figure 6 and Figure 7, it is possible to verify that metabolism and distribution start to increase as in green, the systemic circulation concertation of the drug is lower than the amount dissolute or absorbed, which means metabolism is occurring. Table 7 summarizes the results of CL, Vss, and T_1/2_ with the different tested LogP values and particle size. It is seen that the initial high LogP values demonstrated barely any metabolism or clearance. The LogP of 6 still has a long T_1/2,_ but this is consistent with a study performed in premature neonates that reflects a slow clearance of an IM injection of alpha-tocopherol with a half-life of 44 h. This was the only information found in humans within the literature [61].

**Figure 8:**
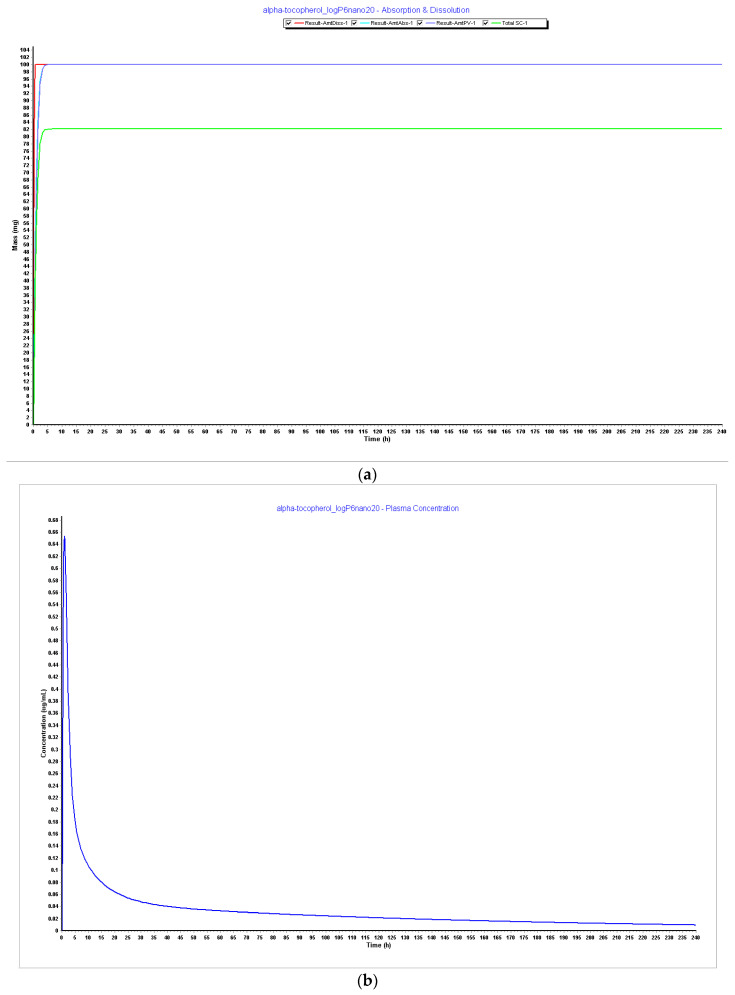

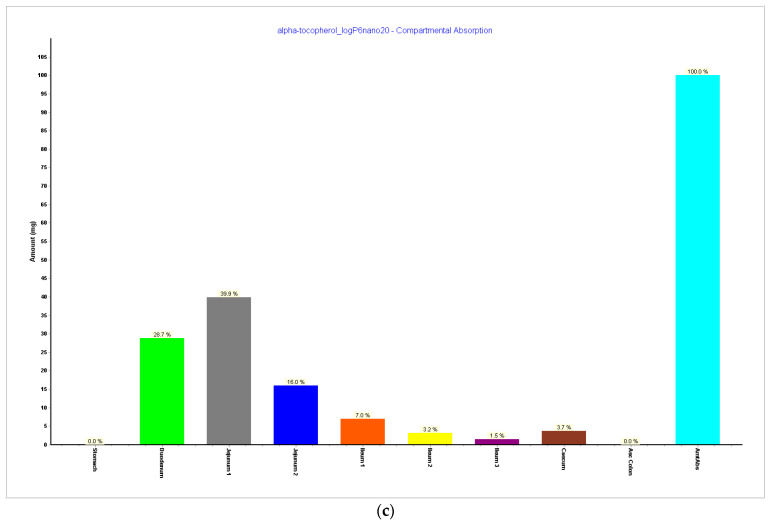
Simulations results during 240 h. (**a**) Absorption and dissolution profile with experimental data, LogP 6, and reduced particle size of 20 nm (radius 10 nm); (**b**) plasma concentration with experimental data, LogP 6, and reduced particle size of 20 nm (radius 10 nm); (**c**) compartmental absorption with experimental data, LogP 6, and reduced particle size of 20 nm (radius 10 nm).

It was also noticed that bioavailability (F%) increased with the increase of LogP and that LogP 6, as described in Figure 8, demonstrates an optimal bioavailability of 82%, which is the reported value in the literature. Although Cmax and Tmax were kept the same, the AUC increased, and when particle size was reduced to 20 nm together with LogP, it did not impact PK parameters. However, since 20 nm particle size for the purpose of oral vaccines is an optimal value, the value of particle size with LogP 6 was considered for the model optimization.

**Figure 9:**
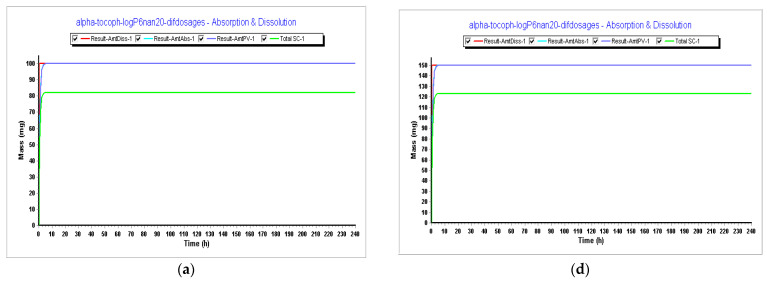

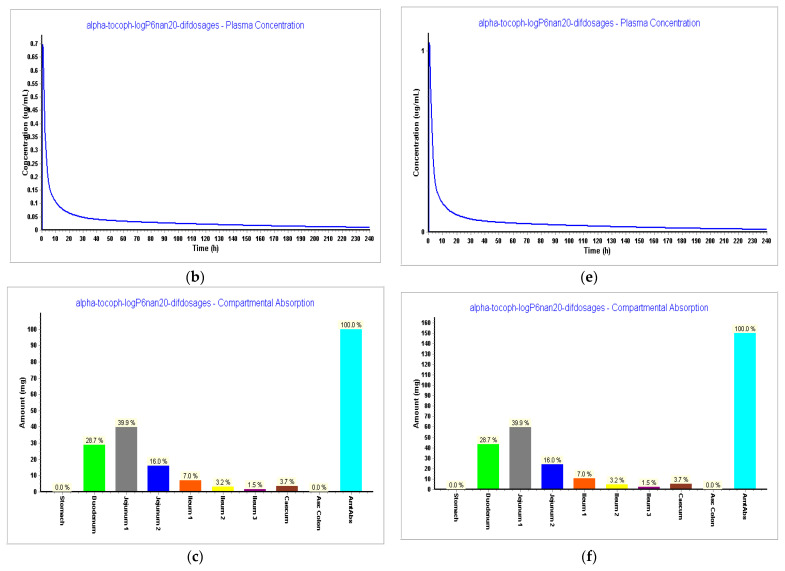
Results during 240 h. (**a**) Absorption and dissolution profile for an IR capsule 100 mg volume 250 mL (0.1 h stomach transit time) with the optimized model from Step 3; (**b**) plasma concentration for an IR capsule 100 mg volume 250 mL (0.1 h stomach transit time) with the optimized model from Step 3; (**c**) compartmental absorption for an IR capsule 100 mg volume 250 mL (0.1 h stomach transit time) with the optimized model from Step 3; (**d**) absorption and dissolution profile for an IR capsule 150 mg volume 200 mL (0.1 h stomach transit time) with the optimized model from Step 3; (**e**) plasma concentration for an IR capsule 150 mg volume 200 mL (0.1 h stomach transit time) with the optimized model from Step 3; (**f**) compartmental absorption for an IR capsule 150 mg volume 200 mL (0.1 h stomach transit time) with the optimized model from Step 3.

**Figure 10:**
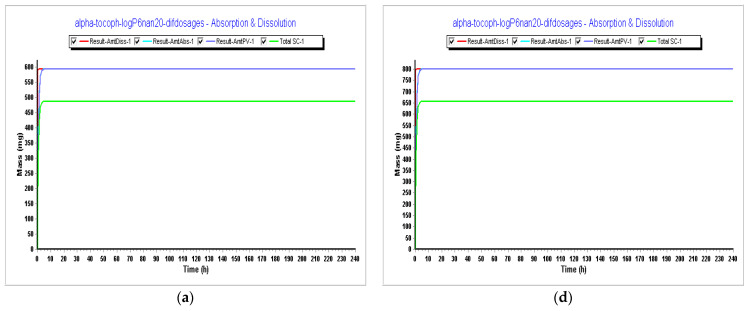

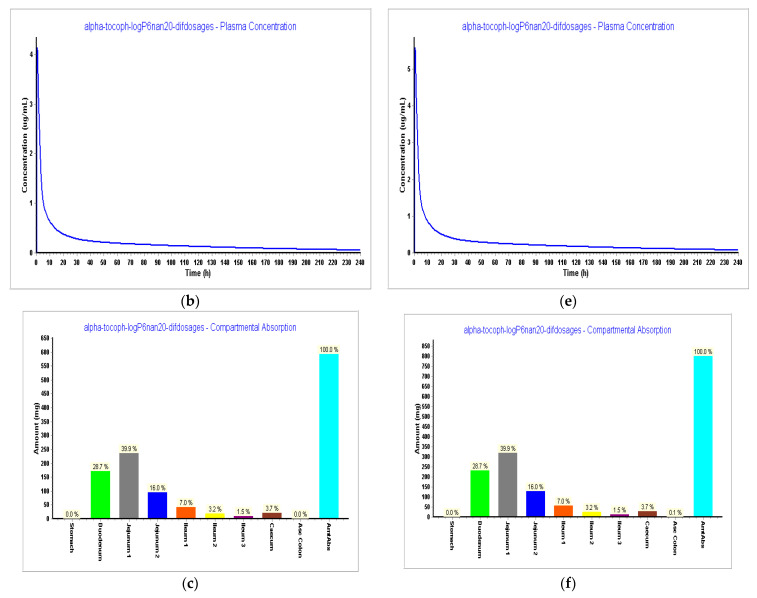
Simulations results during 240 h. (**a**) Absorption and dissolution profile for an IR capsule 593 mg volume 50 mL (0.1 h stomach transit time) with the optimized model from Step 3; (**b**) plasma concentration for an IR capsule 593 mg volume 50 mL (0.1 h stomach transit time) with the optimized model from Step 3; (**c**) compartmental absorption for an IR capsule 593 mg volume 50 mL (0.1 h stomach transit time) with the optimized model from Step 3; (**d**) absorption and dissolution profile for an IR capsule 800 mg volume 68 mL (0.1 h stomach transit time with the optimized model from Step 3; (**e**) plasma concentration for an IR capsule 800 mg volume 68 mL (0.1 h stomach transit time) with the optimized model from Step 3; (**f**) compartmental absorption for an IR capsule 800 mg volume 68 mL (0.1 h stomach transit time) with the optimized model from Step 3.

In Step 4, we tested different dosages to see what dosage of α-tocopherol would better fit in a formulation for an oral vaccine. Tested dosages, as shown in Figure 9 and Figure 10, demonstrated that an AUC_0–inf_ (μg·h/mL) of almost 15 and an AUC_0–t_ (μg·h/mL) of 12.5 was achieved for the 150 mg capsule with a volume of 200 mL. Therefore, this would be the proposed dosage for this indication. When we compare different drugs, AUC can provide insights into their relative bioavailability and overall exposure. A drug with a higher AUC might have better efficacy or a longer duration of action compared to a drug with a lower AUC. Also, higher AUC values might be associated with greater efficacy, but this relationship can be complex and depends on factors like the drug’s mechanism of action, therapeutic range, and safety profile.

This first PBPK model of an oral vaccine aims to verify if α-tocopherol, which is an approved and well-known adjuvant for IM vaccines, could also be endorsed as an adjuvant for an oral vaccine, considering it is a well-known supplement with available formulations worldwide for oral route and also its antioxidant benefits that could boost the immune response together with the antigen.

Since the LogP of the compound was identified as a limitation and to achieve better ADME properties, this LogP had to be decreased to meet optimal conditions of oral drug delivery according to values from the literature. Future research would explore how it is possible to achieve the LogP of 6. The question would be what could be added to the formulation that could lead to this result. Furthermore, it is explored here that for this formulation to be successful, nanoparticles should be used. Another point that could be explored was to perform simulations not only in the fed state but also in the fasted state as a lipidic environment could enhance the solubility of the compound.

## Figures and Tables

**Table 1:** GastroPlus modules used in this study.

Module	Function
ADMET Predictor^®^	Perform predictions from the structure of the compound of physicochemical, pharmacokinetic, and CYP metabolism kinetic parameters required for the GastroPlus PBPK simulations. It is a good tool to use when there is no experimental data to input.
Metabolism and Transporter	Calculates Michaelis–Menten rates for gut and liver (or any PBPK tissue) metabolism and for carrier-mediated transport (influx or efflux—again, for any tissue in a PBPK model) based on experimental data values for Vmax and Km.
PBPKPlus™	Simulate the elimination and distribution of the compound within the body and verify tissue accumulation.

**Table 5:** Results of Steps 1, 2, and 3 (particle size effect).

Parameters	Step 1	Step 2	Step 3
			Particle Size Effect
	Simulation without Experimental Data	Simulation with Experimental Data	20 nm, Radius: 10 nm
Fa (%)	4.9473	100	100
FDp (%)	4.9473	100	100
F (%)	4.9473	99.998	99.998
Cmax (μg/mL)	0.011	0.81464	0.82222
Tmax (h)	2.9	1.1	1.1
AUC 0–inf (μg·h/mL)	14.419	122.88	132.12
AUC 0–t (μg·h/mL)	0.52019	11.782	14.407
CMax Liver (μg/mL)	0.15719	14.563	14.4
Total simulation time (h)	240		

Fa%—shows the net (absorption minus exsorption) percent of the dose that has moved from the lumen into the enterocytes at the time shown above; FDp%—shows the net percent of the dose that has reached the portal vein. In the absence of gut metabolism and drug accumulation in enterocytes, this number will be equal to Fa% (for oral doses); F%—% bioavailable. If there is no liver metabolism for this drug, F% will be equal to FDp% (for oral doses). If the drug does not undergo either one of the processes like gut metabolism, accumulation in gut tissue, or liver metabolism, all three parameters—Fa%, FDp%, and F% will be equal; AUC 0–inf (ng.h/mL)— shows the current value of the area under the central compartment plasma concentration-time curve. This value is AUC extrapolated to infinity; AUC 0–t (ng.h/mL)— shows the current value of the area under the central compartment plasma concentration-time curve. This value is AUC for the time of the simulation—it is not extrapolated to infinity.

**Table 6:** PK parameter results with particle size effect and LogP effect alone and together.

Parameters	Step 3							
	Particle Size Effect	LogP						Particle Size Effect + LogP
	20 nm, Radius: 10 nm	3.5	4	4.5	5	5.5	6	20 nm, Radius: 10 nm LogP 6
Fa (%)	100	99.94	99.998	99.999	100	100	100	100
FDp (%)	100	99.94	99.998	99.999	100	100	100	100
F (%)	99.998	2.032	4.9489	13.125	31.703	59.256	82.096	82.096
Cmax (ug/mL)	0.82222	0.01343	0.03344	0.09031	0.22909	0.4482	0.65365	0.65365
Tmax (h)	1.1	0.6	0.6	0.6	0.7	0.9	1	1
AUC_0–inf_ (μg·h/mL)	132.12	0.04726	0.11853	0.34337	1.0497	3.22	9.7307	9.7307
AUC_0–t_ (μg·h/mL)	14.407	0.04721	0.1185	0.34288	1.044	2.4171	8.3324	8.3324
CMax Liver (μg/mL)	14.4	0.24477	0.5523	1.6393	4.2281	8.3914	11.641	11.641
Total simulation time (h)	240							

**Table 7:** Clearance, distribution volume (Vss), and half-life time (Thal) of alpha-tocopherol through Steps 1–3.

Parameters	Step 1	Step 2	Step 3							
	ADMET Predictor Vales	Experimental Data	Particle Size Effect	LogP						Particle Size Effect + LogP
			20 nm, Radius: 10 nm	3.5	4	4.5	5	5.5	6	20 nm, Radius: 10 nm LogP 6
CL(L/h)	0.242	0.243	0.867	43.129	41.871	38.344	30.330	18.443	8.590	8.590
Vss(L)	3332.433	3337.632	2298.941	639.460	533.859	640.625	730.322	819.876	916.183	916.183
Thal (h)	9548.944	9526.212	1837.160	10.275	8.836	11.578	16.687	30.807	73.915	73.915

**Table 8:** PK parameters after the simulation with different dosages and oral forms.

Parameters	Step 4				
	Dosage and Pharmaceutical Form				
	Tablet 100 mg Volume 250 mL	Capsule 100 mg Volume 250 mL	Capsule 150 mg Volume 200 mL	Capsule 593 mg Volume 50 mL	Capsule 800 mg Volume 68 mL
Fa (%)	100	100	100	100	99.999
FDp(%)	100	100	100	100	99.999
F(%)	82.096	82.096	82.096	82.096	82.096
Cmax (ug/mL)	0.65365	0.69821	1.0473	4.1404	5.5857
Tmax (h)	1	0.8	0.8	0.8	0.8
AUC 0–inf (μg·h/mL)	9.7307	9.7307	14.596	57.703	77.845
AUC 0–t (μg·h/mL)	8.3324	8.3338	12.501	49.42	66.671
CMax Liver (ug/mL)	11.641	12.853	19.28	76.221	102.83
Total simulation time (h)	240				

## Data Availability

Not applicable.

## References

[1] Khakimova A., Abdollahi L., Zolotarev O., Rahim F. (2022). Global interest in vaccines during the COVID-19 pandemic: Evidence from Google Trends. Vaccine X.

[2] Top Pharma Trends to Watch in 2023. https://www.alpha-sense.com/blog/trends/pharma-industry-trends/.

[3] Trends in Global Vaccine Development: 2023 and Beyond. https://www.clinicaltrialsarena.com/sponsored/trends-in-global-vaccine-development-2023-and-beyond/.

[4] 2023 Global Life Sciences Outlook—Innovating and Collaborating for Tomorrow. https://www.deloitte.com/global/en/Industries/life-sciences-health-care/perspectives/global-life-sciences-sector-outlook.html.

[5] Saldanha L., Vale N. (2022). The First Approved COVID-19 Vaccines: The Road to Cancer Vaccines. Int. J. Transl. Med..

[6] AstraZeneca to Set Up Division for Vaccines and Antibody Therapies. https://www.reuters.com/business/healthcare-pharmaceuticals/astrazeneca-create-separate-division-vaccines-antibody-therapies-2021-11-09/.

[7] Moderna to Hire around 2000 Employees Amid mRNA Development Push. https://www.reuters.com/business/healthcare-pharmaceuticals/moderna-hire-around-2000-employees-globally-by-2023-end-2023-03-10/.

[8] Next in Pharma: Thriving in 2023. https://www.pwc.com/us/en/industries/pharma-life-sciences/pharmaceutical-industry-trends.html.

[9] Saldanha L., Langel Ü., Vale N. (2023). In Silico Studies to Support Vaccine Development. Pharmaceutics.

[10] Tegenge M.A., Mitkus R.J. (2015). A first-generation physiologically based pharmacokinetic (PBPK) model of alpha-tocopherol in human influenza vaccine adjuvant. Regul. Toxicol. Pharmacol..

[11] Garçon N., Vaughn D.W., Didierlaurent A.M. (2012). Development and evaluation of AS03, an Adjuvant System containing α-tocopherol and squalene in an oil-in-water emulsion. Expert Rev. Vaccines.

[12] Lodaya R.N., Kanitkar A.P., Friedrich K., Henson D., Yamagata R., Nuti S., Mallett C.P., Bertholet S., Amiji M.M., O’Hagan D.T. (2019). Formulation Design, Optimization and In Vivo Evaluations of an α-Tocopherol-Containing Self-Emulsified Adjuvant System using Inactivated Influenza Vaccine. J. Control. Release.

[13] Das Gupta S., Suh N. (2016). Tocopherols in cancer: An update. Mol. Nutr. Food Res..

[14] Jiang Q. (2017). Natural Forms of Vitamin E as Effective Agents for Cancer Prevention and Therapy. Adv. Nutr..

[15] Cao P., Xu Z.P., Li L. (2022). Tailoring functional nanoparticles for oral vaccine delivery: Recent advances and future perspectives. Compos. Part B Eng..

[16] Miquel-Clopés A., Bentley E.G., Stewart J.P., Carding S.R. (2019). Mucosal vaccines and technology. Clin. Exp. Immunol..

[17] New R.R.C. (2019). Formulation technologies for oral vaccines. Clin. Exp. Immunol..

[18] Vela Ramirez J.E., Sharpe L.A., Peppas N.A. (2017). Current state and challenges in developing oral vaccines. Adv. Drug Deliv. Rev..

[19] Hoft D.F., Brusic V., Sakala I.G. (2011). Optimizing vaccine development. Cell. Microbiol..

[20] Coffey J.W., Gaiha G.D., Traverso G. (2021). Oral Biologic Delivery: Advances Toward Oral Subunit, DNA, and mRNA Vaccines and the Potential for Mass Vaccination During Pandemics. Annu. Rev. Pharmacol. Toxicol..

[21] Lavelle E.C., Ward R.W. (2022). Mucosal vaccines—Fortifying the frontiers. Nat. Rev. Immunol..

[22] Li M., Kaminskas L.M., Marasini N. (2021). Recent advances in nano/microparticle-based oral vaccines. J. Pharm. Investig..

[23] Mitchell M.J., Billingsley M.M., Haley R.M., Wechsler M.E., Peppas N.A., Langer R. (2021). Engineering precision nanoparticles for drug delivery. Nat. Rev. Drug Discov..

[24] Oehlke K., Adamiuk M., Behsnilian D., Gräf V., Mayer-Miebach E., Walz E., Greiner R. (2014). Potential bioavailability enhancement of bioactive compounds using food-grade engineered nanomaterials: A review of the existing evidence. Food Funct..

[25] Samineni R., Chimakurthy J., Konidala S. (2022). Emerging Role of Biopharmaceutical Classification and Biopharmaceutical Drug Disposition System in Dosage form Development: A Systematic Review. Turk. J. Pharm. Sci..

[26] Mardones P., Rigotti A. (2004). Cellular mechanisms of vitamin E uptake: Relevance in alpha-tocopherol metabolism and potential implications for disease. J. Nutr. Biochem..

[27] Nielsen P.B., Müllertz A., Norling T., Kristensen H.G. (2001). The effect of α-tocopherol on the in vitro solubilisation of lipophilic drugs. Int. J. Pharm..

[28] Atkinson J., Manor D., Parker R., Lennarz W.J., Lane M.D. (2013). Vitamin E. Encyclopedia of Biological Chemistry.

[29] (2023). Clinical Evaluation of New Vaccines—Scientific Guideline.

[30] Flory S., Birringer M., Frank J., Weber P., Birringer M., Blumberg J.B., Eggersdorfer M., Frank J. (2019). Bioavailability and Metabolism of Vitamin E. Vitamin E in Human Health.

[31] Novotny Dura J., Fadel J.G., Holstege D.M., Furr H.C., Clifford A.J. (2012). Kinetics, bioavailability, and metabolism of RRR-alpha-tocopherol in humans supports lower requirement for vitamin E. J. Nutr..

[32] Aljurbui S.J., Hussain A., Yusuf M., Ramzan M., Afzal O., Almohaywi B., Yasmin S., Altamimi A.S.A. (2022). Impact of Composition and Morphology of Ketoconazole-Loaded Solid Lipid Nanoparticles on Intestinal Permeation and Gastroplus-Based Prediction Studies. ACS Omega.

[33] GastroPlus^®^ PBBM/PBPK. https://www.simulations-plus.com/software/gastroplus/.

[34] Jones H.M., Gardner I.B., Collard W.T., Stanley P., Oxley P., Hosea N.A., Plowchalk D., Gernhardt S., Lin J., Dickins M. (2011). Simulation of Human Intravenous and Oral Pharmacokinetics of 21 Diverse Compounds Using Physiologically Based Pharmacokinetic Modelling. Clin. Pharmacokinet..

[35] Duquesnoy C., Lacey L.F., Keene O.N., Bye A. (1998). Evaluation of different partial AUCs as indirect measures of rate of drug absorption in comparative pharmacokinetic studies. Eur. J. Pharm. Sci..

[36] Lacey L.F., Keene O.N., Duquesnoy C., Bye A. (1994). Evaluation of different indirect measures of rate of drug absorption in comparative pharmacokinetic studies. J. Pharm. Sci..

[37] Atanasova I., Bozhinova K., Todorova D., Terziivanov D. (2003). Pharmacokinetics and comparative bioavailability of two metformin formulations after single-dose administration in healthy subjects. Clin. Drug Investig..

[38] Marcelín-Jiménez G., Angeles-Moreno A.P., Contreras-Zavala L., Morales-Martínez M., Rivera-Espinosa L. (2009). A single-dose, three-period, six-sequence crossover study comparing the bioavailability of solution, suspension, and enteric-coated tablets of magnesium valproate in healthy Mexican volunteers under fasting conditions. Clin. Ther..

[39] Endrenyi L., Tothfalusi L. (2012). Metrics for the evaluation of bioequivalence of modified-release formulations. AAPS J..

[40] Chen M.L., Davit B., Lionberger R., Wahba Z., Ahn H.Y., Yu L.X. (2011). Using partial area for evaluation of bioavailability and bioequivalence. Pharm. Res..

[41] Perlik V., Kulasekaran A., Coutinho G., Votava M., Cardot J.-M. (2023). Relationship between Pharmacokinetic Profile and Clinical Efficacy Data of Three Different Forms of Locally Applied Flurbiprofen in the Mouth/Throat. Pharmaceutics.

[42] Grabowski T., Jaroszewski J.J., Piotrowski W., Sasinowska-Motyl M. (2014). Method of variability optimization in pharmacokinetic data analysis. Eur. J. Drug Metab. Pharmacokinet..

[43] Fourie Zirkelbach J., Jackson A.J., Wang Y., Schuirmann D.J. (2013). Use of partial AUC (PAUC) to evaluate bioequivalence--a case study with complex absorption: Methylphenidate. Pharm. Res..

[44] Zhang Y., Jiang X.H., Hu Y.Q., Li Z.R., Su L., Wang Z.G., Ma G. (2008). MDR1 genotypes do not influence the absorption of a single oral dose of 600 mg valacyclovir in healthy Chinese Han ethnic males. Br. J. Clin. Pharmacol..

[45] Merck, α-Tocopherol. https://www.sigmaaldrich.com/PT/en/product/sigma/258024.

[46] DL-Alpha-Tocopherol (Compound). https://pubchem.ncbi.nlm.nih.gov/compound/DL-alpha-Tocopherol#section=Refractive-Index.

[47] Compound Alpha-Tocopherol (FDB000565). https://foodb.ca/compounds/FDB000565.

[48] Horiba Scientific. Size Results for Vitamin E. https://www.horiba.com/int/scientific/applications/pharmaceutical/pages/particle-size-analysis-of-vitamins/#:~:text=Size%20Results%20for%20Vitamin%20E&text=The%20reported%20arithmetic%20mean%20on,distribution%20of%20Vitamin%20E%20dispersion.

[49] DrugBank. Vitamin E. https://go.drugbank.com/drugs/DB00163.

[50] Poukka R.K.H., Bieri J.G. (1970). Blood α-tocopherol: Erythrocyte and plasma relationships in vitro and in vivo. Lipids.

[51] Cui A., Xiao P., Fan Z., Zeng Y., Wang H., Zhuang Y. (2023). Associations between vitamin E status and bone mineral density in children and adolescents aged 8-19 years: Evidence based on NHANES 2005–2006, 2017–2018. PLoS ONE.

[52] Chen J., He J., Hamm L., Batuman V., Whelton P.K. (2002). Serum Antioxidant Vitamins and Blood Pressure in the United States Population. Hypertension.

[53] Cole E.T., Cadé D., Benameur H. (2008). Challenges and opportunities in the encapsulation of liquid and semi-solid formulations into capsules for oral administration. Adv. Drug Deliv. Rev..

[54] Ferslew K.E., Acuff R.V., Daigneault E.A., Woolley T.W., Stanton P.E. (1993). Pharmacokinetics and bioavailability of the RRR and all racemic stereoisomers of alpha-tocopherol in humans after single oral administration. J. Clin. Pharmacol..

[55] Desmarchelier C., Borel P. (2017). Bioavailability of Vitamin E. Bioavailability of Vitamin E in Humans.

[56] Saez V., Souza I.D.L., Mansur C.R.E. (2018). Lipid nanoparticles (SLN & NLC) for delivery of vitamin E: A comprehensive review. Int. J. Cosmet. Sci..

[57] Berger T.A., Berger B.K., Kogelman K. (2022). Supercritical Fluid Chromatography for Chiral Analysis and Semi-preparative Purification. Reference Module in Chemistry, Molecular Sciences and Chemical Engineering.

[58] Gao Y., Gesenberg C., Zheng W., Qiu Y., Chen Y., Zhang G.G.Z., Yu L., Mantri R.V. (2017). Chapter 17—Oral Formulations for Preclinical Studies: Principle, Design, and Development Considerations. Developing Solid Oral Dosage Forms.

[59] Lindsley C.W., Stolerman I.P., Price L.H. (2010). Lipophilicity. Encyclopedia of Psychopharmacology.

[60] Stephens C., Lucena M.I., Andrade R.J., McQueen C.A. (2018). 2.26—Idiosyncratic Drug-Induced Liver Injury: Mechanisms and Susceptibility Factors. Comprehensive Toxicology.

[61] Colburn W.A., Ehrenkranz R.A. (1983). Pharmacokinetics of a single intramuscular injection of vitamin E to premature neonates. Pediatr. Pharmacol..

